# Association of the TRIB1 tribbles homolog 1 gene rs17321515 A>G polymorphism and serum lipid levels in the Mulao and Han populations

**DOI:** 10.1186/1476-511X-10-230

**Published:** 2011-12-06

**Authors:** Lynn Htet Htet Aung, Rui-Xing Yin, Dong-Feng Wu, Qing Li, Ting-Ting Yan, Yi-Ming Wang, Hui Li, Dai-Xun Wei, Yuan-Lu Shi, De-Zhai Yang

**Affiliations:** 1 Department of Cardiology, Institute of Cardiovascular Diseases, the First Affiliated Hospital, Guangxi Medical University, 22 Shuangyong Road, Nanning 530021, Guangxi, People's Republic of China; 2 College of Stomatology, Guangxi Medical University, 10 Shuangyong Road, Nanning 530021, Guangxi, People's Republic of China; 3 Clinical Laboratory of the Affiliated Cancer Hospital, Guangxi Medical University, 71 Hedi Road, Nanning 530021, People's Republic of China; 4 The Center for Disease Control and Prevention of Luocheng Mulao Autonomous County, Luocheng 546400, Hechi, Guangxi, People's Republic of China; 5 Department of Molecular Biology, Medical Scientific Research Center, Guangxi Medical University, 22 Shuangyong Road, Nanning 530021, Guangxi, People's Republic of China

## Abstract

**Background:**

The association of rs17321515 single nucleotide polymorphism (SNP) near TRIB1 gene and serum lipid profiles has never been studied in the Chinese population. Therefore, the present study was undertaken to detect the association of rs17321515 SNP and several environmental factors on serum lipid levels in the Mulao and Han populations.

**Methods:**

A total of 639 unrelated subjects of Mulao nationality and 644 participants of Han nationality were randomly selected from our previous stratified randomized cluster samples. Genotypes of the TRIB1 rs17321515 A>G SNP were determined via polymerase chain reaction and restriction fragment length polymorphism, and then confirmed by direct sequencing.

**Results:**

Serum apolipoprotein (Apo) B levels were higher in Mulao than in Han (*P *< 0.05). There were no differences in the genotypic and allelic frequencies between the two ethnic groups (*P *> 0.05). High- and low-density lipoprotein cholesterol (HDL-C and LDL-C) levels in Han were different among the genotypes (*P *< 0.05 for each), the subjects with AG/GG genotypes had higher HDL-C and LDL-C levels than the subjects with AA genotype. Total cholesterol (TC), HDL-C, LDL-C, ApoA1 and ApoB levels in Han males were different among the genotypes (*P *< 0.05-0.001), the G carriers had higher TC, HDL-C, LDL-C, ApoA1 and ApoB levels than the G noncarriers. HDL-C levels in Mulao males were different among the genotypes (*P *< 0.05), the G carriers had lower HDL-C levels than the G noncarriers. Serum HDL-C and LDL-C levels in both ethnic groups and TG levels in Han were correlated with the genotypes or alleles (*P *< 0.05-0.01). TG and HDL-C levels in Mulao males and TG, HDL-C, LDL-C and ApoA1 levels in Han males were correlated with genotypes or alleles (*P *< 0.05-0.001). TG and ApoA1 levels in Han females were associated with genotypes (*P *< 0.05 for each). Serum lipid parameters were also associated with several environmental factors in both ethnic groups.

**Conclusions:**

The associations of TRIB1 rs17321515 SNP and serum lipid levels are different between the Mulao and Han populations. These discrepancies might partly result from different TRIB1 gene-environmental interactions in both ethnic groups.

## Introduction

Over decades, it has evident that the most attributable and heritable risk factor for coronary artery disease (CAD) is the unfavorable serum lipid profile such as elevated serum levels of total cholesterol (TC) [1], triglyceride (TG) [2], low-density lipoprotein cholesterol (LDL-C) [3], and apolipoprotein (Apo) B [4], or low levels of high-density lipoprotein cholesterol (HDL-C) [5] and ApoA1 [4]. As a result, serum lipid level is the major modifiable risk factor for CAD and also the main target for therapeutic intervention [6]. Disorders of lipid metabolism are well recognized as a complex trait caused by multiple environmental and genetic factors [7-9] and their interactions [10,11]. It is well known that almost 40-60% of the inter-individual variation in plasma lipid phenotypes can be explained by genetic polymorphisms [12-14].

It is noticeable that through genome-wide association studies, a growing number of new loci involving in lipid metabolism have been identified [15-17]. The Tribbles, drosophila, Homolog of, 1 (TRIB1) (http://www.ncbi.nlm.nih.gov/gene) is one of the potential candidate genes that play a substantial role in the cholesterol metabolism and the atherosclerosis process [18-20]. It is located on chromosome 8q24 [15,17,21] and encodes the protein tribbles homolog 1, which is a member of the recently identified tribbles protein family, governing as an adaptor or scaffold protein [22]. One study demonstrating the role of TRIB1 in lipoprotein metabolism in mice reported that TRIB1 overexpression significantly reduced the plasma levels of very low-density lipoprotein (VLDL), LDL-C, HDL-C and TG; TRIB1 deficiency experienced the reverse effects on plasma cholesterol and TG in vise versa [23]. Recently, a common single nucleotide polymorphism (SNP) adjacent to the TRIB1 gene locus namely rs17321515 is a very compelling SNP effecting lipoprotein metabolism. The minor allele at this SNP was found to be associated with an atheroprotective lipid phenotype of lower TG and LDL-C, and higher HDL-C resulting in a significantly reduced risk of CAD in European [7], Malays and Asian Indians [24]. This association was also genome-wide significant in severe hypertriglyceridemic subjects [25]. However, the effect of this SNP on serum lipid levels was not functionally validated and the mechanism was yet unclear. Furthermore, the reproducibility of this association has not been detected in the Chinese population so far.

Among a total of 56 ethnic groups in China, Han is the largest one. Mulao (also known as Mulam) is one of the minorities with a population of 207,352 according to the fifth national census statistics of China in 2000. Ninety percent of them live in the Luocheng Mulao Autonomous County, Guangxi Zhuang Autonomous Region. They call themselves "Ling" and a smaller group call themselves "Jin" or "Bendiren". Historical data show that the history of this ethnic minority can be traced back to the Jin Dynasty (AD 265-420). It is believed that the Mulao people are the descendants of the ancient Baiyue tribe in south China and ethnically related to the neighboring ethnic groups. One previous study has shown that the genetic relationship between Mulao nationality and other minorities in Guangxi was much closer than that between Mulao and Han or Uighur nationality [26]. In a previous study, we have shown a significant association of UDP-N-acetyl-alpha-D-galactosamine: polypeptide N-acetylgalactosaminyltransferase 2 gene (*GALNT2*) polymorphisms and serum lipid levels in the Mulao population [27]. To the best of our knowledge, however, no prior study has been conducted about the association of rs17321515 SNP and serum lipid levels in the Chinese population. Therefore, the aim of the present study was to assess the association of TRIB1 gene rs17321515 SNP and several environmental factors with serum lipid phenotypes in the Mulao and Han populations.

## Materials and methods

### Study population

A total of 639 unrelated subjects of Mulao nationality comprising 301 males (47.1%) and 338 females (52.9%) and 644 unrelated participants of Han nationality including 299 men (46.4%) and 345 women (53.6%) were randomly selected from our previous stratified randomized cluster samples. All of them were rural agricultural workers residing in Luocheng Mulao Autonomous County, Guangxi Zhuang Autonomous Region, People's Republic of China. The age range was from 15 to 80 years. The mean age of Mulao participants was 53.11 ± 14.97 years, whereas that of Han subjects was 53.32 ± 15.14 years. All participants were essentially healthy and had no evidence of diseases related to atherosclerosis, CAD and diabetes. Any participant had a history of taking medications known to affect serum lipid levels (lipid-lowering drugs such as statins or fibrates, beta-blockers, diuretics, or hormones) was excluded before the blood sample was taken. The study design was approved by the Ethics Committee of the First Affiliated Hospital, Guangxi Medical University. Informed consent was obtained from all participants.

### Epidemiological survey

The survey was carried out using internationally standardized methods, following a common protocol [28]. Information on demographics, socioeconomic status, and lifestyle factors was collected with standardized questionnaires. The intake of alcohol was quantified as the number of liangs (about 50 g) of rice wine, corn wine, rum, beer, or liquor consumed during the preceding 12 months. Alcohol consumption was categorized into groups of grams of alcohol per day: < 25 and ≥ 25. Smoking status was categorized into groups of cigarettes per day: < 20 and ≥ 20. In the physical examination, several parameters such as height, weight, and waist circumference were measured. Sitting blood pressure was measured three times with the use of a mercury sphygmomanometer after a 5-minute of rest, and the average of the three measurements was recorded. Systolic blood pressure was determined by the first Korotkoff sound, and diastolic blood pressure by the fifth Korotkoff sound. Body weight, to the nearest 50 grams, was measured using a portable balance scale. Subjects were weighed in a minimum of clothing with shoes off. Height was measured, to the nearest 0.5 cm, using a stadiometer. From these two measurements body mass index (BMI, kg/m^2^) was calculated.

### Biochemical measurements

A venous blood sample of 5 mL was obtained from all subjects after at least 12 hours of fasting. A two fifth of the sample (2 mL) was collected into glass tubes and used to determine serum lipid levels. The remaining three fifth of the sample (3 mL) was transferred to the tubes contained anticoagulants (4.80 g/L citric acid, 14.70 g/L glucose, and 13.20 g/L tri-sodium citrate) and was used to extract deoxyribonucleic acid (DNA). Measurements of serum TC, TG, HDL-C, and LDL-C levels in the samples were performed by enzymatic methods with commercially available kits (RANDOX Laboratories Ltd., Ardmore, Diamond Road, Crumlin Co. Antrim, United Kingdom, BT29 4QY; Daiichi Pure Chemicals Co., Ltd., Tokyo, Japan). Serum ApoA1 and ApoB levels were detected by the immunoturbidimetric immunoassay using a commercial kit (RANDOX Laboratories Ltd.). All determinations were performed with an autoanalyzer (Type 7170A; Hitachi Ltd., Tokyo, Japan) in the Clinical Science Experiment Center of the First Affiliated Hospital, Guangxi Medical University [29,30].

### DNA amplification and genotyping

Genomic DNA was isolated from peripheral blood leukocytes using the phenol-chloroform method [31,32]. The extracted DNA was stored at 4°C until analysis. Genotyping of the TRIB1 rs17321515 SNP was performed by polymerase chain reaction and restriction fragment length polymorphism (PCR-RFLP). PCR amplification was performed using 5'-AGTGCAGCAAAGTGGAAAGAG-3' and 5'-AGAGCGAGACTGTCACACACA-3' (Sangon, Shanghai, People's Republic of China) as the forward and reverse primer pairs; respectively. Each amplification reaction was performed in a total volume of 25 mL, containing 10 × PCR buffer (1.8 mM MgCl_2_) 2.5 μL, 1 U *Taq *polymerase, 2.5 mmol/L of each dNTP (Tiangen, Beijing, People's Republic of China) 2.0 μL, 20 pmol/L of each primer and 50 ng of genomic DNA, processing started with 95 °C for 5 min and followed by 45 s of denaturing at 94 °C, 45 s of annealing at 60 °C and 1 min of elongation at 72 °C for 33 cycles. The amplification was completed by a final extension at 72 °C for 7 min. Then 10 U of *Eco130I *(*StyI*) enzyme was added directly to the PCR products (10 μL) and digested at 37 °C overnight. After restriction enzyme digestion of the amplified DNA, genotypes were identified by electrophoresis on 2% ethidium-bromide stained agarose gels and visualizing with ultraviolet illumination. Genotypes were scored by an experienced reader blinded to the epidemiological and lipid results. Twelve samples (AA, AG and GG genotypes in four; respectively) detected by the PCR-RFLP were also confirmed by direct sequencing. The PCR product was purified by low melting point gel electrophoresis and phenol extraction, and then the DNA sequences were analyzed in Shanghai Sangon Biological Engineering Technology & Services Co., Ltd., People's Republic of China.

### Diagnostic criteria

The normal values of serum TC, TG, HDL-C, LDL-C, ApoA1 and ApoB levels, and the ratio of ApoA1 to ApoB in our Clinical Science Experiment Center were 3.10-5.17, 0.56-1.70, 1.16-1.42, 2.70-3.10 mmol/L, 1.20-1.60, 0.80-1.05 g/L, and 1.00-2.50; respectively. The individuals with TC > 5.17 mmol/L and/or TG > 1.70 mmol/L were defined as hyperlipidemic [29,30]. Hypertension was diagnosed according to the criteria of 1999 World Health Organization-International Society of Hypertension Guidelines for the management of hypertension [33,34]. The diagnostic criteria of overweight and obesity were according to the Cooperative Meta-analysis Group of China Obesity Task Force. Normal weight, overweight and obesity were defined as a BMI < 24, 24-28, and > 28 kg/m^2^; respectively [29-34].

### Statistical analyses

Epidemiological data were recorded on a pre-designed form and managed with Excel software. Data analysis was performed using the software SPSS version 19.0 (SPSS Inc., Chicago, Illinois). Quantitative variables were expressed as mean ± standard deviation (serum TG levels were presented as medians and interquartile ranges). Qualitative variables were expressed as percentages. Allele frequency was determined via direct counting, and the standard goodness-of-fit test was used to test the Hardy-Weinberg equilibrium. Difference in genotype distribution between the groups was obtained using the chi-square test. The difference in general characteristics between Mulao and Han was tested by the Student's unpaired *t*-test. The association of genotypes and serum lipid parameters was tested by analysis of covariance (ANCOVA). Sex, age, BMI, blood pressure, alcohol consumption, cigarette smoking were adjusted for the statistical analysis. Multivariate linear regression analysis with stepwise modeling was performed to evaluate the association of serum lipid levels with genotypes (AA = 1, AG = 2 and GG = 3) and several environment factors in the combined population of Mulao and Han, Mulao, Han, males and females; respectively. A *P *value of less than 0.05 was considered statistically significant.

## Results

### General characteristics and serum lipid levels

The comparison of general characteristics and serum lipid levels between the Mulao and Han populations is summarized in Table 1. The levels of body weight, BMI, diastolic blood pressure were significantly lower in Mulao than in Han (*P *< 0.05-0.001), whereas the level of ApoB was higher in Mulao than in Han (*P *< 0.001). There were no significant differences in the levels of systolic blood pressure, serum TC, TG, HDL-C, LDL-C, ApoA1, the ratio of ApoA1 to ApoB, age structure, gender ratio, height, the percentage of smoking or the percentage of alcohol consumption between the two ethnic groups (*P *> 0.05 for all).

**Table 1:** Comparison of demography, lifestyle and serum lipid levels between the Mulao and Han populations

Characteristics	Mulao	Han	*t *(χ^2^)	*P*
Number	639	644	-	-
Male/female	301/338	299/345	-0.183	0.855
Age (years)	53.11 ± 14.97	53.32 ± 15.14	-0.314	0.754
Height (cm)	155.38 ± 8.23	154.70 ± 8.11	1.507	0.132
Weight (kg)	52.88. ± 9.56	54.10 ± 9.16	-2.369	0.018
Body mass index (kg/m^2^)	21.84 ± 3.16	22.61 ± 3.58	-4.102	0.000
Systolic blood pressure (mmHg)	130.18 ± 22.00	130.25 ± 18.68	-0.089	0.929
Diastolic blood pressure (mmHg)	81.14 ± 11.28	82.81 ± 11.10	-2.664	0.008
Pulse pressure (mmHg)	49.04 ± 16.65	47.44 ± 13.95	1.822	0.069
Blood glucose level (mmol/L)	6.04 ± 1.58	6.09 ± 1.68	-0.498	0.619
Cigarette smoking [n (%)]				
Nonsmoker	464 (72.6)	455 (70.7)		
<20 cigarettes/day	133 (20.8)	137 (21.3)		
≥20 cigarettes/day	42 (6.5)	52 (8.1)	-0.027	0.978
Alcohol consumption [n (%)]				
Nondrinker	478 (74.8)	485 (75.3)		
<25 g/day	54 (8.5)	68 (10.6)		
≥25 g/day	107 (16.7)	91 (14.1)	1.823	0.069
Total cholesterol (mmol/L)	5.06 ± 1.36	5.07 ± 1.12	-0.015	0.988
Triglyceride (mmol/L)	1.07(0.78)	1.09(0.96)	-0.926	0.354
HDL-C (mmol/L)	1.76 ± 0.46	1.72 ± 0.55	-1.114	0.265
LDL-C (mmol/L)	2.94 ± 0.91	2.93 ± 0.89	0.290	0.772
Apolipoprotein (Apo) A1 (g/L)	1.33 ± 0.41	1.34 ± 0.27	-0.360	0.719
ApoB (g/L)	0.97 ± 0.52	0.87 ± 0.21	4.378	0.000
Apo A1/ApoB	1.59±0.71	1.62 ± 0.50	-0.983	0.326

TC, total cholesterol; TG, triglyceride; HDL-C, high-density lipoprotein cholesterol; LDL-C, low-density lipoprotein cholesterol; ApoA1, apolipoprotein A1; ApoB, apolipoprotein B.

The values of triglyceride were presented as median (interquartile range). The difference between the two ethnic groups was determined by the Wilcoxon-Mann-Whitney test.

### Results of electrophoresis and genotyping

**Figure 1:**
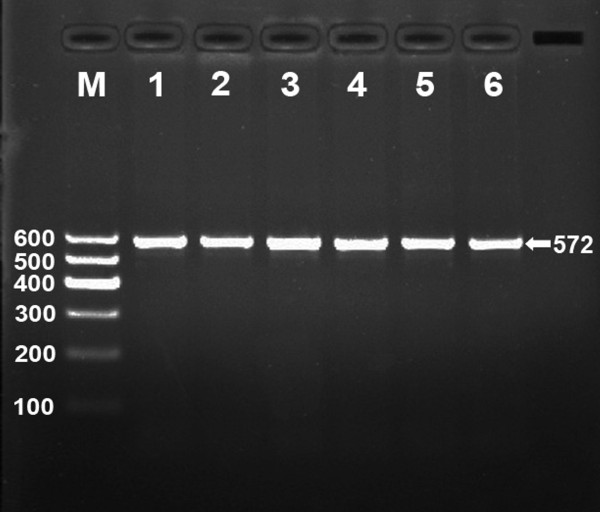
**Electrophoresis of PCR products of the samples**. Lane M, 100 bp marker ladder; lanes 1-6, samples. The 572 bp bands are the target genes.

**Figure 2:**
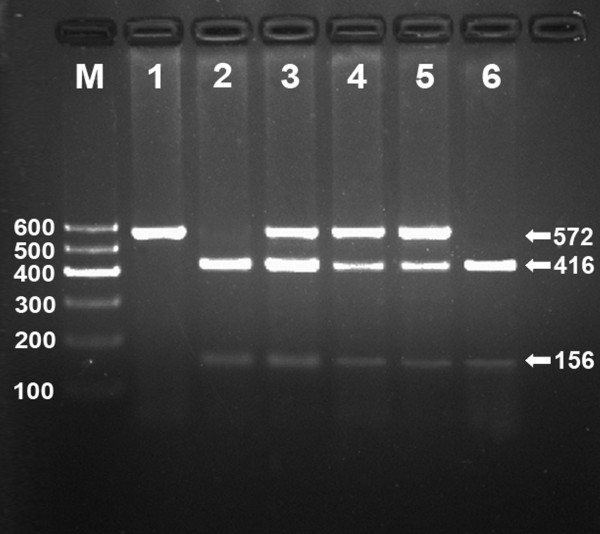
**Genotyping of the TRIB1 rs17321515 SNP**. Lane M, 100 bp marker ladder; lane 1, GG genotype (572 bp); lanes 2 and 6, AA genotype (416- and 156-bp); and lanes 3, 4 and 5, AG genotype (572-, 416- and 156-bp).

After the genomic DNA of the samples was amplified by PCR and imaged by 2% agarose gel electrophoresis, the products of 572 bp nucleotide sequences were found in all samples (Figure 1). The genotypes identified were named according to the presence or absence of the enzyme restriction sites, whether A or G nucleotide at rs17321515 locus of the TRIB1 gene. The presence of the cutting site indicated the A allele, while its absence indicated the G allele (cannot be cut). Thus, the GG genotype was homozygote for the absence of the site (band at 572 bp), AG genotype was heterozygote for the presence and absence of the site (bands at 572-, 416- and 156-bp), and AA genotype was homozygote for the presence of the site (bands at 416- and 156-bp; Figure 2).

### Genotypic and allelic frequencies

The genotypic and allelic frequencies of rs17321515 SNP in the TRIB1 gene are shown in Table 2. The frequencies of A and G alleles were 51.5% and 48.5% in Mulao; and 50.0% each in Han (*P *> 0.05). The frequencies of AA, AG and GG genotypes were 24.3%, 54.5% and 21.3% in Mulao, and 28.7%, 48.5% and 22.8% in Han (*P *> 0.05); respectively. A significant difference in the genotypic frequencies was detected between Han males and females (*P *< 0.05) although the allelic frequencies were insignificantly different (*P *> 0.05). But there was no significant difference in the genotypic and allelic frequencies between Mulao males and females (*P *> 0.05 for each).

**Table 2:** Comparison of the genotypic and allelic frequencies between the Mulao and Han populations [n (%)]

Group	n	Genotype			Allele	
		AA	AG	GG	A	G
Mulao	639	155(24.3)	348(54.5)	136(21.2)	658(51.5)	620(48.5)
Han	644	185(28.7)	312(48.5)	147(22.8)	682(50.0)	682(50.0)
χ^2^	-	5.019			0.782	
*P*	-	0.081			0.376	
Mulao						
Male	301	80(26.6)	155(51.5)	66(21.9)	315(52.3)	287(47.7)
Female	338	75(22.2)	193(57.1)	70(20.7)	343(50.7)	333(49.3)
χ^2^	-	2.662			0.554	
*P*	-	0.264			0.457	
Han						
Male	299	73(24.4)	160(53.5)	66(22.1)	306(51.2)	292(48.8)
Female	345	112(32.5)	152(44.0)	81(23.5)	376(54.5)	314(45.5)
χ^2^	-	6.706			1.419	
*P*	-	0.035			0.234	

### Results of sequencing

**Figure 3:**
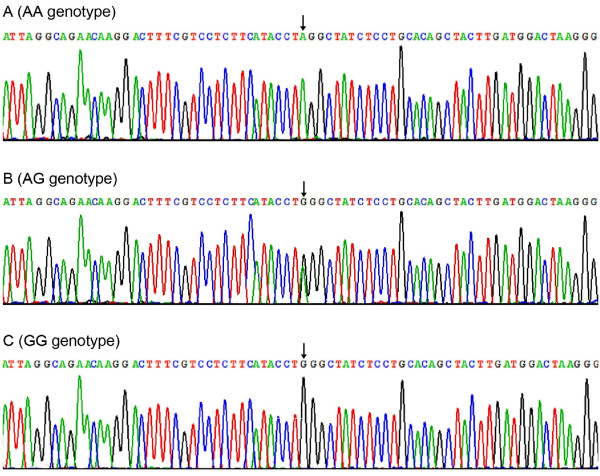
**A part of the nucleotide sequence of the TRIB1 rs17321515 SNP**. (A) AA genotype; (B) AG genotype; (C) GG genotype.

The results were shown as AA, AG and GG genotypes by PCR-RFLP, the AA, AG and GG genotypes were also confirmed by sequencing (Figure 3); respectively.

### Genotypes and serum lipid levels

As shown in Table 3, the levels of HDL-C and LDL-C in Han but not in Mulao were different among the AA, AG and GG genotypes (*P *< 0.05 for each), the subjects with AG/GG genotypes had higher serum HDL-C and LDL-C levels than the subjects with AA genotype. Subgroup analyses showed that the levels of TC, HDL-C, LDL-C, ApoA1 and ApoB in Han males but not in females were different among the three genotypes (*P *< 0.05-0.001), the G carriers had higher serum TC, HDL-C, LDL-C, ApoA1 and ApoB levels than the G noncarriers. Besides, the levels of HDL-C in Mulao males were different among the three genotypes (*P *< 0.05), the G carriers had lower serum HDL-C levels than the G noncarriers.

**Table 3:** Comparison of serum lipid levels among the genotypes between the Mulao and Han populations

Genotype	n	TC (mmol/L)	TG (mmol/L)	HDL-C (mmol/L)	LDL-C (mmol/L)	ApoA1 (g/L)	ApoB (g/L)	ApoA1/ ApoB
Mulao								
AA	155	5.13 ± 1.07	1.10(0.75)	1.80 ± 0.54	3.05 ± 0.88	1.34 ± 0.43	1.00 ± 0.61	1.61 ± 0.85
AG	348	5.05 ± 1.28	1.04(0.79)	1.75 ± 0.44	2.93 ± 0.91	1.35 ± 0.39	0.96 ± 0.49	1.60 ± 0.63
GG	136	5.02 ± 1.82	1.09(0.90)	1.71 ± 0.41	2.84 ± 0.93	1.27 ± 0.43	0.94 ± 0.49	1.54 ± 0.71
*F*	-	0.283	1.492	2.316	1.882	2.073	0.450	0.396
*P*	-	0.754	0.474	0.099	0.153	0.127	0.638	0.673
AA	155	5.13 ± 1.07	1.10(0.75)	1.82 ± 0.54	3.05 ± 0.88	1.34 ± 0.43	1.00 ± 0.61	1.61 ± 0.85
AG/GG	484	5.05 ± 1.44	1.05(0.80)	1.74 ± 0.43	2.91 ± 0.92	1.33 ± 0.40	0.96 ± 0.49	1.58 ± 0.65
*F*	-	0.410	-0.904	3.700	2.754	0.052	0.754	0.208
*P*	-	0.522	0.366	0.055	0.097	0.819	0.385	0.648
Male								
AA	80	5.13 ± 0.86	1.09(1.20)	1.84 ± 0.63	2.99 ± 0.69	1.37 ± 0.44	1.04 ± 0.67	1.59 ± 0.80
AG	155	5.22 ± 1.30	1.19(1.11)	1.76 ± 0.45	2.91 ± 0.87	1.39 ± 0.39	0.98 ± 0.49	1.58 ± 0.59
GG	66	5.11 ± 2.30	1.29(1.12)	1.63 ± 0.40^a^	2.75 ± 0.97	1.28 ± 0.48	1.03 ± 0.61	1.45 ± 0.68
*F*	-	0.154	3.403	3.652	1.510	1.577	0.358	1.017
*P*	-	0.858	0.182	0.027	0.223	0.208	0.700	0.363
Female								
AA	75	5.14 ± 1.26	1.19(0.72)	1.79 ± 0.42	3.09 ± 1.05	1.30 ± 0.41	0.96 ± 0.51	1.62 ± 0.90
AG	193	4.92 ± 1.25	1.00(0.64)	1.74 ± 0.44	2.96 ± 0.95	1.32 ± 0.38	0.94 ± 0.50	1.62 ± 0.66
GG	70	4.93 ± 1.17	1.03(0.60)	1.77 ± 0.41	2.91 ± 0.88	1.25 ± 0.39	0.86 ± 0.35	1.63 ± 0.73
*F*	-	0.954	2.297	0.325	0.686	0.714	1.006	0.005
*P*	-	0.386	0.317	0.723	0.505	0.491	0.367	0.995
Han								
AA	185	4.97 ± 1.18	1.23(1.07)	1.63 ± 0.41	2.79 ± 0.85	1.32 ± 0.25	0.86 ± 0.18	1.62 ± 0.54
AG	312	5.11 ± 1.10	1.08(1.01)	1.78 ± 0.65^a^	3.00 ± 0.87^a^	1.35 ± 0.25	0.87 ± 0.21	1.63 ± 0.46
GG	147	5.10 ± 1.11	1.00(0.70)	1.73 ± 0.48	2.95 ± 0.96	1.33 ± 0.31	0.88 ± 0.24	1.61 ± 0.55
*F*	-	0.926	6.130	4.275	3.359	0.975	0.458	0.112
*P*	-	0.397	0.047	0.014	0.035	0.378	0.633	0.894
AA	185	4.99 ± 1.18	1.23(1.06)	1.64 ± 0.41	2.79 ± 0.85	1.33 ± 0.25	0.86 ± 0.18	1.62 ± 0.54
AG/GG	459	5.10 ± 1.10	1.05(0.95)	1.76 ± 0.60	2.98 ± 0.90	1.34 ± 0.27	0.87 ± 0.22	1.62 ± 0.49
*F*	-	1.403	-1.756	6.598	6.749	0.609	0.507	0.002
*P*	-	0.237	0.079	0.010	0.010	0.435	0.477	0.963
Male								
AA	73	4.98 ± 1.29	1.30(0.93)	1.53 ± 0.38	2.62 ± 0.75	1.27 ± 0.26	0.88 ± 0.19	1.49 ± 0.47
AG	160	5.35 ± 1.12^a^	1.30(1.10)	1.69 ± 0.40^a^	3.14 ± 0.83^a^	1.37 ± 0.27^a^	0.94 ± 0.21	1.53 ± 0.42
GG	66	5.41 ± 1.05^a^	1.15(1.16)	1.74 ± 0.47^a^	3.14 ± 0.90^a^	1.41 ± 0.33^a^	0.97 ± 0.24^a^	1.52 ± 0.49
*F*	-	3.178	0.329	5.594	10.859	5.919	3.302	0.234
*P*	-	0.043	0.848	0.004	0.000	0.003	0.038	0.791
Female								
AA	112	4.94 ± 1.10	1.15(1.09)	1.72 ± 0.41	2.89 ± 0.90	1.35 ± 0.25	0.83 ± 0.17	1.72 ± 0.56
AG	152	4.87 ± 1.04	0.97(0.81)	1.85 ± 0.83	2.84 ± 0.89	1.33 ± 0.24	0.82 ± 0.20	1.72 ± 0.47
GG	81	4.86 ± 1.09	0.95(0.60)	1.72 ± 0.49	2.82 ± 0.98	1.28 ± 0.29	0.80 ± 0.21	1.68 ± 0.59
*F*	-	0.199	4.704	2.020	0.190	1.903	0.461	0.171
*P*	-	0.820	0.095	0.134	0.827	0.151	0.631	0.843

TC, total cholesterol; TG, triglyceride; HDL-C, high-density lipoprotein cholesterol; LDL-C, low-density lipoprotein cholesterol; ApoA1, apolipoprotein A1; ApoB, apolipoprotein B. The values of triglyceride were presented as median (interquartile range). The difference among the genotypes was determined by the Kruskal-Wallis test or the Wilcoxon-Mann-Whitney test. ^a^*P *< 0.05 comparison with AA genotype of the same ethnic group.

### Relative factors for serum lipid parameters

The multiple linear regression analysis showed that the levels of serum HDL-C and LDL-C were correlated with the genotypes or alleles in both ethnic groups. The levels of TG in Han was also correlated with the genotypes (*P *< 0.05-0.01; Table 4).

**Table 4:** Relative risk factors for serum lipid parameters in the Mulao and Han populations

Lipid parameter	Risk factor	Unstandardized coefficient	Standard error	Standardized coefficient	*t*	*P*
Mulao/Han						
TC	Alcohol comsumption	0.232	0.047	0.137	4.892	0.000
	Body mass index	0.045	0.010	0.121	4.324	0.000
	Age	0.097	0.025	0.107	3.875	0.000
TG	Gender	-0.517	0.162	-0.105	-3.199	0.001
	Alcohol comsumption	0.491	0.093	0.148	5.287	0.000
	Body mass index	0.095	0.020	0.132	4.720	0.000
	Blood glucose level	0.145	0.042	0.096	3.475	0.001
HDL-C	Body mass index	-0.030	0.004	-0.200	-7.136	0.000
	Alcohol comsumption	0.079	0.019	0.115	4.103	0.000
	Gender	0.144	0.034	0.140	4.298	0.000
LDL-C	Body mass index	0.042	0.007	0.159	5.720	0.000
	Age	0.083	0.018	0.127	4.589	0.000
ApoA1	Alcohol comsumption	0.116	0.013	0.249	8.995	0.000
	Body mass index	-0.009	0.003	-0.088	-3.171	0.002
	Gender	0.053	0.023	0.077	2.355	0.019
ApoB	Body mass index	0.021	0.003	0.175	6.325	0.000
	Ethnic group	-0.112	0.022	-0.139	-5.070	0.000
	Blood glucose level	0.026	0.007	0.104	3.800	0.000
	Gender	-0.084	0.022	-0.104	-3.778	0.000
ApoA1/ApoB	Body mass index	-0.042	0.005	-0.240	-8.851	0.000
	Blood glucose level	-0.045	0.010	-0.123	-4.446	0.000
	Alcohol comsumption	0.132	0.026	0.162	5.051	0.000
	Gender	0.193	0.038	0.160	5.011	0.000
	Age	-0.030	0.012	-0.069	-2.510	0.012
Mulao						
TC	Body mass index	0.062	0.017	0.143	3.665	0.000
	Alcohol consumption	0.196	0.070	0.110	2.800	0.005
	Age	0.081	0.038	0.083	2.135	0.033
TG	Alcohol consumption	0.548	0.110	0.193	4.991	0.000
	Body mass index	0.107	0.026	0.156	4.032	0.000
HDL-C	Body mass index	-0.038	0.006	-0.258	-6.793	0.000
	Alcohol consumption	0.126	0.028	0.207	4.536	0.000
	Gender	0.099	0.042	0.107	2.339	0.020
	Genotype	-0.052	0.026	-0.075	-1.970	0.049
LDL-C	Body mass index	0.054	0.011	0.188	4.830	0.000
	Alcohol consumption	-0.117	0.047	-0.098	-2.525	0.012
	Age	0.059	0.025	0.091	2.353	0.019
	Genotype	-0.105	0.053	-0.077	-1.979	0.048
ApoA1	Alcohol consumption	0.120	0.021	0.225	5.822	0.000
	Blood glucose level	-0.020	0.010	-0.077	-1.996	0.046
ApoB	Body mass index	0.028	0.006	0.167	4.264	0.000
ApoA1/ApoB	Body mass index	-0.044	0.009	-0.198	-5.154	0.000
	Blood glucose level	-0.052	0.017	-0.117	-3.046	0.002
	Alcohol consumption	0.158	0.043	0.172	3.723	0.000
	Gender	0.170	0.065	0.121	2.622	0.009
Han						
TC	Alcohol consumption	0.175	0.071	0.111	2.449	0.015
	Diastolic blood pressure	0.020	0.004	0.193	4.949	0.000
	Gender	-0.237	0.103	-0.105	-2.309	0.021
	Blood glucose level	0.080	0.026	0.119	3.070	0.002
TG	Gender	-0.948	0.215	-0.173	-4.405	0.000
	Blood glucose level	0.245	0.065	0.151	3.759	0.000
	Diastolic blood pressure	0.038	0.010	0.155	3.800	0.000
	Age	-0.241	0.083	-0.122	-2.915	0.004
	Genotype	-0.368	0.151	-0.095	-2.442	0.015
HDL-C	Body mass index	-0.021	0.006	-0.135	-3.331	0.001
	Alcohol consumption	0.133	0.036	0.172	3.668	0.000
	Gender	0.207	0.052	0.185	3.960	0.000
	Allele	0.120	0.050	0.096	2.412	0.016
LDL-C	Body mass index	0.038	0.010	0.154	3.932	0.000
	Age	0.093	0.026	0.144	3.560	0.000
	Allele	0.209	0.078	0.104	2.666	0.008
	Blood glucose level	0.054	0.021	0.103	2.544	0.011
ApoA1	Alcohol consumption	0.111	0.015	0.297	7.561	0.000
	Body mass index	-0.013	0.003	-0.178	-4.526	0.000
ApoB	Body mass index	0.014	0.002	0.241	6.551	0.000
	Blood glucose level	0.033	0.004	0.263	7.435	0.000
	Gender	-0.076	0.017	-0.181	-4.370	0.000
	Diastolic blood pressure	0.003	0.001	0.140	3.843	0.000
	Alcohol consumption	0.027	0.012	0.094	2.243	0.025
ApoA1/ApoB	Body mass index	-0.039	0.005	-0.292	-7.634	0.000
	Blood glucose level	-0.050	0.010	-0.175	-4.766	0.000
	Alcohol consumption	0.113	0.029	0.170	3.889	0.000
	Gender	0.212	0.041	0.222	5.141	0.000
	Diastolic blood pressure	-0.005	0.002	-0.116	-3.064	0.002

TC, total cholesterol; TG, triglyceride; HDL-C, high-density lipoprotein cholesterol; LDL-C, low-density lipoprotein cholesterol; ApoA1, apolipoprotein A1; ApoB, apolipoprotein B.

As shown in Table 5, when the lipid data were analyzed according to gender, the levels of TG and HDL-C in Mulao were correlated with genotypes in males but not in females (*P *< 0.05 for each). The levels of TG, HDL-C, LDL-C and ApoA1 in Han were associated with genotypes or alleles in males, and the levels of TG and ApoA1 were associated with genotypes in females (*P *< 0.05-0.001).

**Table 5:** Correlative factors for serum lipid parameters between males and females in both ethnic groups

Lipid parameter	Risk factor	Unstandardized coefficient	Standard error	Standardized coefficient	*t*	*P*
Mulao						
Male						
TC	Body mass index	0.061	0.027	0.128	2.225	0.027
	Alcohol consumption	0.205	0.095	0.124	2.161	0.031
TG	Body mass index	0.165	0.054	0.172	3.044	0.003
	Alcohol consumption	0.468	0.189	0.140	2.475	0.014
	Genotype	0.519	0.246	0.119	2.110	0.036
HDL-C	Body mass index	-0.041	0.009	-0.256	-4.679	0.000
	Alcohol consumption	0.135	0.030	0.244	4.459	0.000
	Genotype	-0.098	0.039	-0.135	-2.485	0.014
ApoA1	Alcohol consumption	0.143	0.026	0.305	5.579	0.000
	Blood glucose level	-0.032	0.013	-0.135	-2.473	0.014
ApoB	Body mass index	0.021	0.011	0.114	1.984	0.048
ApoA1/ApoB	Alcohol consumption	0.167	0.041	0.225	4.094	0.000
	Body mass index	-0.049	0.012	-0.232	-4.228	0.000
	Blood glucose level	-0.057	0.020	-0.152	-2.768	0.006
Female						
TC	Body mass index	0.061	0.021	0.157	2.926	0.004
	Age	0.122	0.047	0.138	2.571	0.011
TG	Body mass index	0.053	0.013	0.212	3.970	0.000
HDL-C	Body mass index	-0.036	0.007	-0.267	-5.074	0.000
LDL-C	Body mass index	0.071	0.016	0.235	4.470	0.000
	Age	0.099	0.036	0.144	2.742	0.006
ApoA1	Diastolic blood pressure	0.004	0.002	0.120	2.213	0.028
ApoB	Body mass index	0.031	0.008	0.210	3.956	0.000
	Age	0.036	0.018	0.108	2.039	0.042
ApoA1/ApoB	Body mass index	-0.042	0.012	-0.182	-3.420	0.001
	Age	-0.075	0.028	-0.144	-2.708	0.007
Han						
Male						
TC	Alcohol consumption	0.180	0.077	0.132	2.339	0.020
	Diastolic blood pressure	0.027	0.006	0.265	4.697	0.000
	Blood glucose level	0.077	0.037	0.115	2.056	0.041
TG	Body mass index	0.165	0.054	0.172	3.044	0.003
	Alcohol consumption	0.468	0.189	0.140	2.475	0.014
	Genotype	0.519	0.246	0.119	2.110	0.036
HDL-C	Body mass index	-0.027	0.006	-0.261	-4.721	0.000
	Alcohol consumption	0.129	0.027	0.263	4.759	0.000
	Allele	0.176	0.055	0.174	3.180	0.002
	Blood glucose level	-0.028	0.013	-0.117	-2.150	0.032
LDL-C	Allele	0.563	0.118	0.271	4.781	0.000
	Systolic blood pressure	0.005	0.003	0.120	2.106	0.036
	Blood glucose level	0.058	0.028	0.119	2.103	0.036
ApoA1	Alcohol consumption	0.134	0.018	0.405	7.529	0.000
	Body mass index	-0.015	0.004	-0.213	-3.955	0.000
	Genotype	0.063	0.023	0.149	2.796	0.006
ApoB	Body mass index	0.009	0.003	0.185	3.341	0.001
	Diastolic blood pressure	0.004	0.001	0.217	3.920	0.000
	Blood glucose level	0.033	0.006	0.279	5.291	0.000
	Alcohol consumption	0.035	0.013	0.142	2.647	0.009
ApoA1/ApoB	Alcohol consumption	0.098	0.028	0.188	3.479	0.001
	Body mass index	-0.039	0.006	-0.361	-6.708	0.000
	Blood glucose level	-0.057	0.014	-0.223	-4.184	0.000
Female						
TC	Body mass index	0.061	0.019	0.174	3.285	0.001
	Age	0.209	0.042	0.266	5.028	0.000
TG	Body mass index	0.083	0.020	0.218	4.117	0.000
	Genotype	-0.184	0.083	-0.118	-2.228	0.027
	Blood glucose level	0.135	0.037	0.190	3.620	0.000
	Diastolic blood pressure	0.016	0.006	0.147	2.800	0.005
LDL-C	Body mass index	0.062	0.016	0.205	3.859	0.000
	Age	0.204	0.036	0.303	5.725	0.000
	Alcohol consumption	-0.533	0.199	-0.144	-2.675	0.008
ApoA1	Genotype	-0.047	0.019	-0.136	-2.471	0.014
ApoA1/ApoB	Alcohol consumption	0.305	0.107	0.153	2.851	0.005
	Body mass index	-0.047	0.009	-0.292	-5.510	0.000
	Age	-0.082	0.019	-0.227	-4.287	0.000

TC, total cholesterol; TG, triglyceride; HDL-C, high-density lipoprotein cholesterol; LDL-C, low-density lipoprotein cholesterol; ApoA1, apolipoprotein A1; ApoB, apolipoprotein B.

Serum lipid parameters were also associated with age, gender, BMI, systolic and diastolic blood pressure, fasting blood glucose levels and alcohol consumption in both ethnic groups (*P *< 0.05-0.001, Tables 4 and 5).

## Discussion

By comparison of serum lipid levels between the participants of Mulao and Han nationalities, we showed that the levels of ApoB were higher in Mulao than in Han (*P *< 0.05). There were no significant differences in the levels of TC, HDL-C, LDL-C, ApoA1, and the ratio of ApoA1 to ApoB between the two ethnic groups. These findings are not fully consistent with those of our recent study [27]. In the previous study, we showed that both LDL-C and ApoB levels were higher in Mulao than in Han [27]. This discrepancy may be related to the different sampling from our previous stratified randomized cluster samples. It is well recognized that serum lipid levels are influenced by both environmental factors including demographics, diet, alcohol consumption, cigarette smoking, obesity, exercise, hypertension [8,9] and genetic factors such as variants in genes coding for proteins functioning lipid metabolism; and their interactions [10,11] as well as ethnic diversities. Mulao people have been abided with their special customs especially in case of marriage. Their marriages were family-arranged since childhood. However, divorce and remarriage were also allowed. Although the marriage was arranged at childhood, it was celebrated only when the girl reached puberty. Traditionally, until before a Mulao girl delivered her first child, she stayed with her parents and was unrestricted to join the young people social activities such as responsive singing, flirtations, and courtships at festival times. The age of the wife was usually four or five years older than that of the husband. Engagement and marriage were socially accepted with bride-wealth payment. Interestingly, there was a preference of marriage to cousins from maternal side (mother's brother's daughter). Therefore, it is compelling us to believe that some hereditary characteristics and genotypes of lipid metabolism-related genes in this population may be different from those in Han nationality [35-38].

The genotypic and allelic frequencies of TRIB1 rs17321515 SNP in different studies were slightly dissimilar. In a genome-wide association study of nearly 10000 participants of European ancestry and Asians, the frequencies of A and G alleles were 55.0% and 45.0% [15], which is similar to those of British population [17]. Another study detecting the polygenic determinants of severe hypertriglyceridemia found that the frequencies of AA, AG and GG genotypes were 42.4%, 47.0% and 10.6% in hypertriglyceridemic patients and 28.2%, 47.6% and 24.2% in normal controls [25]. In a Spanish familial hypercholesterolemia cohort study, the frequencies of AA, AG and GG genotypes were 30.3%, 51.0% and 19.6%; respectively [39]. Hegele *et al*. [40] reported that the percentages of A and G alleles were 51-68% and 32-49% in classical Fredrickson hyperlipoproteinemic patients. The frequencies of A and G alleles were 48.0% and 52.0% in Japanese population [41], which was similar to those in Malay population [24]. The minor allele frequency was 44.0% in Korean population which is similar to that of European ancestry. In our present study, the frequency of G allele was 48.5% in Mulao and 50.0% in Han, which were closer to that of Japanese and Asian Malay populations. There was no significant difference in both genotypic and allelic frequencies between the Mulao and Han populations. We felt that biologically, Mulao and Han nationalities might be homologous. Upon considering the gender, the genotypic frequency in Han but not in Mulao was different between males and female (*P *< 0.05). These results indicate that the genotype distribution of TRIB1 rs17321515 SNP may have a sex specificity.

Many genome-wide studies have reported that the TRIB1 rs17321515 SNP was associated with variation in serum levels of LDL-C, HDL-C and TG. Kathiresan *et al*. [15] found that the A allele was associated with increased levels of TG and LDL-C and decreased levels of HDL-C in European Ancestry. Tai *et al*. [24] also reported that the A allele was associated with increased levels of TC and LDL-C but not with HDL-C in Malay population. Besides, Tai *et al*. [24] and Willer *et al*. [17] stated that the presence of rs17321515 SNP was also associated with an increased risk for prevalent CAD (OR 1.23 for each copy of the A allele). Recently, Nakayama *et al*. [41] showed that rs17321515 SNP was associated with serum TG (3.5 mg/dL decrease per minor A allele) and LDL-C levels (1.7 mg/dL decrease per minor A allele) in Japanese population. The Spanish familial hypercholesterolemic cohort study concluded that homozygous for A allels in smoker displayed higher plasma TG, higher VLDL-C concentrations and higher TC/HDL-C ratio than carriers of the minor allele G and homozygous AA with the persence of arcus cornealis displayed lower plasma ApoA1 levels and higher TC/HDL-C than AG/GG subjects [39]. A recent study in Korean population also showed rs17321515 SNP was associated with increased serum levels of TG, LDL-C and HDL-C [42]. In the present study, we also found that the levels of HDL-C and LDL-C in Han were different among the AA, AG and GG genotypes, the subjects with AG/GG genotypes had higher serum HDL-C and LDL-C levels than the subjects with AA genotype. The levels of TC, HDL-C, LDL-C, ApoA1 and ApoB in Han males were different among the three genotypes, the G carriers had higher serum TC, HDL-C, LDL-C, ApoA1 and ApoB levels than the G noncarriers. The levels of HDL-C in Mulao males were different among the three genotypes, the G carriers had lower serum HDL-C levels than the G noncarriers. Therefore, the effects of rs17321515 SNP on serum lipid levels might be different between genders although it was not relevant in other studies. Another fact was the mean age of our study populaiton was higher than the other studies. In additions, different environmental and genetic factors might also contribute to variable levels of association with serum lipid levels in different populations.

It is well known that environmental factors such as dietary patterns, lifestyle, obesity, physical activity, and hypertension are all strongly related with serum lipid levels [8,9]. In the present study, we also showed that serum lipid parameters were correlated with age, sex, alcohol consumption, cigarette smoking, BMI, fasting blood glucose levels, and blood pressure in both ethnic groups. These data suggested that the environmental factors also played an important role in determining serum lipid levels in our populations. Although rice and corn are the staple foods in both ethnic groups, Mulao people prefer to eat cold foods along with acidic and spicy dishes, so bean soy sauce and pickled vegetables are among their most popular dishes. They also favor to eat animal offals which contain abundant saturated fatty acid. It has been widely accepted that high-fat diets, particularly those containing large quantities of saturated fatty acids raise blood cholesterol concentrations and predispose individuals to CAD [43]. Many studies also stated that diatery habits can impart a strong influence on serum levels of ApoB, ApoA1 and their ratio, and which in turn can effect the risk of CAD [44-46]. In the current study, we found that the levels of ApoB was higher in Mulao than in Han. This might be partly due to the difference in dietary habit between the Mulao and Han populations.

## Conclusion

The present study showed that the genotypic and allelic frequencies of TRIB1 rs17321515 SNP were not different between the Mulao and Han populations, but the genotypic frequecies were different between Han males and females. The subjects with AG/GG genotypes in Han had higher serum HDL-C and LDL-C levels than the subjects with AA genotype. The G carriers in Han males had higher serum TC, HDL-C, LDL-C, ApoA1 and ApoB levels than the G noncarriers, but the G carriers in Mulao males had lower serum HDL-C levels than the G noncarriers. These differences in the association of TRIB1 rs17321515 SNP and serum lipid profiles between the two ethnic groups might partly result from different TRIB1 gene-enviromental interactions.

## Competing interests

The authors declare that they have no competing interests.

## Authors' contributions

LHHA participated in the design, undertook genotyping, carried out the epidemiological survey, and drafted the manuscript. RXY conceived the study, participated in the design, carried out the epidemiological survey, collected the samples, and helped to draft the manuscript. DFW, QL and TTY collaborated to the genotyping. DFW, YMW, HL, DXW, YLS and DZY carried out the epidemiological survey and collected the samples. All authors read and approved the final manuscript.
